# Utilization and quality of palliative care in patients with hematological and solid cancers: a population-based study

**DOI:** 10.1007/s00432-024-05721-6

**Published:** 2024-04-12

**Authors:** Cordula Gebel, Bianka Ditscheid, Franziska Meissner, Ekaterina Slotina, Isabel Kruschel, Ursula Marschall, Ullrich Wedding, Antje Freytag

**Affiliations:** 1 Department of Palliative Care, University Hospital Jena, Friedrich-Schiller University Jena, Jena, Germany; 2 Institute of General Practice, University Hospital Jena, Friedrich-Schiller University Jena, Jena, Germany; 3 BARMER, Wuppertal, Germany; 4 Comprehensive Cancer Center Central Germany (CCCG), Jena, Deutschland

**Keywords:** Palliative care, Solid tumor, Hematologic cancer, End of life, Quality of healthcare, Claims data

## Abstract

**Background:**

Palliative care (PC) contributes to improved end-of-life care for patients with hematologic malignancies (HM) and solid tumors (ST) by addressing physical and psychological symptoms and spiritual needs. Research on PC in HM vs. ST patients is fragmented and suggests less use.

**Methods:**

We analyzed claims data of all deceased members of a large German health insurance provider for the year before death. First, we analyzed the frequency and the beginning of different types of PC and compared patients with HM vs. ST. Second, we analyzed the adjusted impact of PC use on several end-of-life quality outcomes in patients with HM vs. ST. We performed simple and multiple (logistic) regression analysis, adjusted for relevant covariates, and standardized for age and sex.

**Results:**

Of the 222,493 deceased cancer patients from 2016 to 2020, we included 209,321 in the first analysis and 165,020 in the second analysis. Patients with HM vs. ST received PC less often (40.4 vs. 55.6%) and later (34 vs. 50 days before death). PC use significantly improved all six quality indicators for good end-of-life care. HM patients had worse rates in five of the six indicators compared with ST patients. Interaction terms revealed that patients with ST derived greater benefit from PC in five of six quality indicators than those with HM.

**Conclusion:**

The data highlight the need to integrate PC more often, earlier, and more effectively into the care of patients with HM.

**Supplementary Information:**

The online version contains supplementary material available at 10.1007/s00432-024-05721-6.

## Introduction

Integration of palliative care (PC) into the care of patients with cancer has the potential to improve the management of physical and psychological symptoms, reduce hospital admissions, and increase the quality of end-of-life care (Haun et al. 2017). The use of PC at the end of life for cancer patients is associated with a reduction in the use of high-cost, intensive services (De Palma et al. 2018; Elliott et al. 2021). This is evident for both solid tumor (ST) and hematologic malignancy (HM) patients (Elliott et al. 2021). Despite these findings, patients with HM receive PC less frequently and at a later stage compared to patients with ST (El-Jawahri et al. 2020; Howell et al. 2010b; LeBlanc et al. 2013).

Several trials report, that patients with HM have significant PC needs and often similar or greater end-of-life symptoms than patients with ST (Hochman et al. 2018; Moreno-Alonso et al. 2018). Recent studies of end-of-life care for patients with HM show that they receive more severe treatments than patients with ST, which can cause significant morbidity and distress (Beaussant et al. 2018; Egan et al. 2020a; Hui et al. 2014). Patients with HM also undergo more intensive care unit and inpatient treatments (Egan et al. 2020a; El-Jawahri et al. 2020; Kirtane et al. 2018) and have a higher likelihood of dying in the hospital (Egan et al. 2020a; Howell et al. 2010a).

Analysis of previous studies has shown that the research landscape is fragmented. Some studies are from single medical centers (Allende-Pérez et al. 2023; Burstein et al. 2023; Cheng et al. 2015; Dasch et al. 2017; Eichenauer et al. 2019; Hui et al. 2014; Ishida et al. 2019; Pasquarella et al. 2022), which may limit the generalizability of their findings. Occasionally, investigations are limited to specific patient populations, such as hospitalized patients (Beaussant et al. 2018; Dasch et al. 2017; Salas et al. 2022) or emergency department patients (Verhoef et al. 2020), or individual disease groups such as leukemia (Cheng et al. 2015; Salas et al. 2022) or multiple myeloma (Odejide et al. 2018). Many studies focus on specific types of PC, such as the utilization of hospice services (Egan et al. 2020a; LeBlanc et al. 2013) or specialized palliative home care (Ishida et al. 2019) or inpatient PC only (Eichenauer et al. 2019; Pasquarella et al. 2022). In conclusion, the scientific literature contains some studies that address the differences in PC in patients with ST and HM. However, few data are available on how the utilization of different types of PC differs between patients with ST vs. HM, and how the use of PC affects the quality of end-of-life care in patients with ST vs. HM. Our search revealed that no comprehensive study on this topic has yet been conducted in Germany. We aimed to fill this gap, as it is important to gather evidence from different health systems and collect population-based comprehensive data.

In two analysis, we compare patients with ST and HM to answer the following research questions:

1. How do utilization rates and average time from the beginning of PC to death differ between patients with ST and HM according to different types of PC?
2. What is the impact of received PC on the quality of end-of-life care and how does this impact differ between patients with ST and HM?

## Methods

We took a population-based approach using nationwide claims data from BARMER, a major German health insurance provider, that covers approximately 10 percent of all persons with statutory health insurance in Germany (Krankenkassen.net 2024). The dataset is part of the *pallCompare* project [German Clinical Trials Register (DRKS): DRKS00024133] (Ditscheid et al. 2023; Freytag et al. 2023). The dataset, spanning 2016 to 2020, included pseudonymized demographic information, utilization rates, and time from the beginning of PC to patient death for different types of PC and end-of-life outcomes. We focused on patients aged 19 years or older, with at least 1 year of BARMER insurance before death, residing in Germany, and with at least one coded cancer diagnosis (ICD-10: C00-C97). Two distinct groups were created to compare patients with solid tumors (ICD-10: C00-C80, C97) and hematological malignancies (ICD-10: C81-C96). Patients with both types of diagnoses were excluded (Beaussant et al. 2018).

In Germany, if indicated by the attending physician, every insured person has the right to receive PC. These services are fully covered by public and private health insurance plans. The following types of PC were analyzed: primary palliative care (PPC) is appropriate for the majority of people facing advanced life-limiting illnesses and is provided by general practitioners, specialists, mobile care services, nursing homes, and hospitals (Stichling et al. 2020). Specialised palliative care (SPC) involves the care of patients by dedicated multidisciplinary teams and treats patients with complex needs who require more intensive care. In the outpatient setting, this responsibility is typically assumed by specialist palliative home care (SPHC), which is provided by specialized teams consisting of palliative care physicians and specialized nurses with expertise in PC. Specialized Inpatient PC (SPIC) includes both care provided in PC units and support provided by PC advisory teams to healthcare professionals in hospital wards. In addition, inpatient hospice care, a 24 h nursing care in specialized institutions, was included in SPC. The SPC variable requires at least one claim within the SPHC, SPIC, or inpatient hospice care domains. The total PC variable requires at least one claim for any type of PC. For complete definitions, see Ditscheid et al. (2023).

To reduce the potential effects of confounding variables, certain models included covariates as adjustment factors. These covariates consisted of age, sex, the Charlson Comorbidity Index (CCI) as a measure of aggregated morbidity (Charlson et al. 1987), nursing care dependency (a dichotomous variable indicating its presence), county rurality, and year of death. The variable "county rurality" indicates the proportion of rural residents in the county where the deceased lived. It was taken from the publicly available Indicators and Maps of Spatial and Urban Development (INKAR). This ratio varies between 0 and 1, with high values indicating a high degree of rurality. Previous studies suggested that these variables are correlated with utilization rates and outcomes of end-of-life care (Beaussant et al. 2018; Ditscheid et al. 2023; Egan et al. 2020a, 2020b; Hui et al. 2014; Jackson et al. 2023; Krause et al. 2021; LeBlanc et al. 2018; Rao et al. 2020; Salas et al. 2022).

We standardized all results by age and gender at the level of federal states as the distribution of BARMER decedents is uneven across Germany (Ditscheid et al. 2023). We followed the STROSA and RECORD recommendations for conducting and reporting (Benchimol et al. 2016; Swart et al. 2016).

To address the research questions, we conducted two analyses. For statistical analyses, we used R (Version 4.1.2) and the ‘survey’ package (Lumley 2023). Significance level was set to 5%.

### Analysis 1: PC utilization and timing in patients with ST and HM

Utilization rates of different PC types are based on the presence of at least one billed service during the patients last year of life. The use of billing codes to identify services has been previously used in other studies using claims data (Dasch et al. 2017; Ditscheid et al. 2023; Krause et al. 2021; Radbruch et al. 2015).

For the analysis of PC utilization rates, two logistic regression models were conducted. Model 1, a simple logistic regression, assessed HM/ST group differences. To control for other influences on the outcome, Model 2 employed a multiple logistic regression with groups HM/ST and the covariates. We report descriptive utilization rates as well as odds ratios (OR) and *p* values for ST vs. HM differences from both models.

To address the question of group differences regarding the time interval between the beginning of PC and the patient death, we first calculated the average time from the beginning of PC to patient death and then ran two linear regression models (Model 1: simple linear regression, Model 2: multiple linear regression with the aforementioned covariates). We report regression coefficients (b) and *p* values for ST vs. HM differences from both models. The regression coefficient b can be interpreted as the (adjusted) time difference between the ST and HM groups.

### Analysis 2: Association of PC on the quality of end-of-life care in patients with HM and ST

To answer the second research question, we selected a subset of the population from Analysis 1. Assuming that some time may be required for PC to have an effect, we excluded patients for whom PC services started less than before the period of 30 days before death (Krause et al. 2021; Rao et al. 2020). Therefore, there were two groups regarding PC: (1) No palliative care (noPC) which included individuals without billed PC services in the last year of life. (2) PC that included decedents who received PC at least 30 days before death. Within the predictor noPC/PC, noPC is coded as the reference category. Patient groups ST and HM from analysis 1 were also included in this analysis. Within the predictor ST/HM, ST is coded as the reference category.

Indicators of the quality of end-of-life care are based on relevant studies, primarily focusing on Salas' research on acute leukemia (Salas et al. 2022). These indicators were adapted and supplemented by relevant studies from the German-speaking region (Dasch et al. 2017; Freytag et al. 2023; Krause et al. 2021) and internationally (Beaussant et al. 2018; Earle et al. 2005; Egan et al. 2020b; Hui et al. 2014; Luta et al. 2015; Martins-Branco et al. 2020; Odejide et al. 2016).

The indicators used and how they are defined are listed below.

- Place of death hospital: The category was captured through the type of discharge "Death" in the hospital case data (including deaths during inpatient PC). All other places of death were classified as "outside the hospital" (including deaths at home, in nursing homes, inpatient hospice, or other "domestic" locations).
- Hospitalization: Initiation of hospital treatment during the final 30 days before death, excluding hospital instances involving inpatient PC.
- Intensive care treatment: At least one instance of inpatient intensive care treatment within the final 30 days before death.
- Emergency medical services: At least one instance of emergency medical services utilized within the final 30 days before death.
- Chemotherapy in the last 14 days: At least one occurrence of chemotherapy administered within the last 14 days before death.
- Intensive medical care (Composite score): Intensive medical care is defined by the occurrence of at least one of the following indicators in the last 30 days of life: (1) Insertion or change of a PEG (percutaneous endoscopic gastrostomy), (2) Parenteral nutrition, (3) Tracheostomy, (4) Dialysis, (5) Resuscitation, (6) Mechanical ventilation/insertion of an endotracheal tube.

These quality indicators, based on claims data, relate specifically to the last 30 days of an individual’s life, except chemotherapy, for which we considered the last 14 days of life, in line with previous research (Earle et al. 2005; Hui et al. 2014). All indicators are dichotomous, with 0 indicating absence and 1 indicating presence.

In Analysis 2, we analyzed each of these quality indicators in a multiple logistic regression model using the aforementioned covariates and the following predictors: ST/HM, noPC/PC, and their interaction. The interaction term was included to analyze potential variations in the relationship of noPC/PC and the quality indicators between patients with ST and patients with HM, respectively.

We report OR and *p* values. The rates for the quality indicators (adjusted for the covariates) are presented in Fig. 2. Those adjusted rates (marginal predictive means) were calculated using the svypredmeans function from the R Package survey (Lumley 2023).

## Results

### Analysis 1: PC utilization and timing in patients with ST and HM

**Fig. 1:**
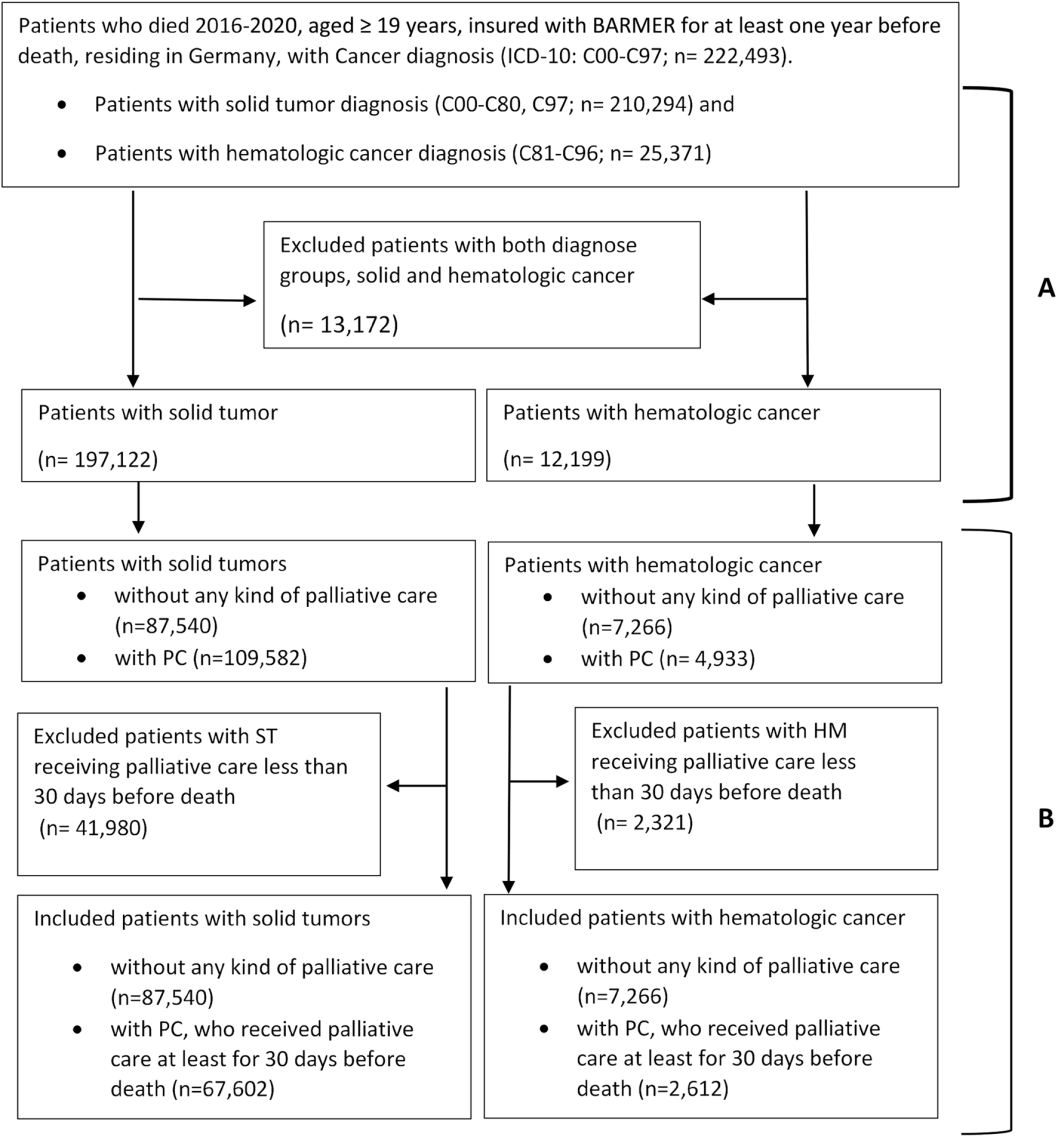
Flowchart Analysis 1 (**A**) and Analysis 2 (**B**). Numbers presented in this figure are based on standardized data. Due to rounding, the sum may not precisely correspond to the provided totals

As shown in Fig. 1A, of the 222,493 individuals who died with a cancer diagnosis between 2016 and 2020, 197,122 patients with ST and 12,199 patients with HM were included. Table 1 summarizes the sample characteristics from analysis 1. Patients with HM were slightly older than those with ST. There were also more women in the HM group and they had a lower CCI score. In addition, fewer patients with HM had a nursing care dependency at the end of life.

**Table 1:** Sample characteristics analysis 1

Characteristic		ST *N* = 197,122	HM *N* = 12,199	Overall *N* = 209,321
Age (years)	Mean (SD)	77.23 (11.87)	77.95 (11.49)	77.28 (11.85)
Female	*n* (%)	88,747 (45.02%)	5882 (48.22%)	94,629 (45.21%)
Charlson Comorbidity Index (CCI)	Mean (SD)	9.37 (3.76)	6.50 (2.82)	9.20 (3.77)
Nursing care dependency at the time of death	*n* (%)	148,964 (75.57%)	8353 (68.47%)	157,317 (75.16%)
County rurality	Mean (SD)	0.19 (0.26)	0.19 (0.26)	0.19 (0.26)
Main diagnosis	*n* (%)	Breast cancer 25,407 (12.89%) Colorectal cancer 28,106 (14.26%) Gastrointestinal cancer 61,024 (30.96%) Lung cancer 32,562 (16.52%) Prostate cancer 28,978 (14.70%)	Non-Hodgkin’s lymphoma 4187 (34.32%) Acute leukemia 2512 (20.59%) Multiple myeloma 2460 (20.17%) Chronic lymphoid leukemia 2152 (17.64%) Chronic myeloid leukemia 882 (7.23%) Hodgkin’s lymphoma 578 (4.74%)	

There may be more than one main diagnosis per person in the claims data. HM diagnosis groups formed according to Beaussant et al. (2018)

#### PC utilization rates

Table 2 shows that 55.6% of patients with ST and 40.4% of patients with HM received PC in the last year of life. The difference between HM and ST was less pronounced for PPC (OR = 0.59) compared to SPC (OR = 0.50).

**Table 2:** Palliative care utilization rates in the last year of life

Group	ST	HM	Statistics Model 1		Statistics Model 2	
	*n* (%)	*n* (%)	OR	*p*	OR	*p*
Total palliative care (PC)	109,582 (55.59%)	4933 (40.44%)	0.54	<0.001	0.78	<0.001
Primary palliative care (PPC)	76,392 (38.75%)	3299 (27.05%)	0.59	<0.001	0.77	<0.001
Specialized palliative care (SPC)	80,133 (40.65%)	3107 (25.47%)	0.50	<0.001	0.73	<0.001
Specialized palliative home care (SPHC)	53,959 (27.37%)	1830 (15.00%)	0.47	<0.001	0.64	<0.001
Specialized palliative inpatient care (SPIC)	39,883 (20.23%)	1525 (12.50%)	0.56	<0.001	0.88	<0.001
Inpatient hospice care	15,146 (7.68%)	388 (3.18%)	0.39	<0.001	0.55	<0.001

PC types are not distinct, which means that a person might have utilized multiple PC types during his/her last year of life. *n* (%) as descriptive utilization rates per group, *OR* odds ratio. Model 1 is a simple logistic regression. Model 2 includes age, sex, CCI, nursing care dependency, county rurality, year of death as covariates. Supplement Table S1 shows all OR including covariate statistics for model 2

A more detailed analysis of SPC showed that inpatient hospice care had the lowest rate of utilization among both groups of patients. Looking at the difference between ST and HM, the OR for inpatient hospice care was 0.39, which was the largest relative difference between the two patient groups. The second largest difference was for SPHC, with an OR of 0.47, where 27% of patients with ST used this form of care compared to 15% of patients with HM.

After adjustment for covariates in Model 2, there were no fundamental changes in the conclusions regarding utilization rates. The OR were generally higher, but still significantly below 1, suggesting that patients with HM were less represented in the different types of PC compared to patients with ST. Further statistical details are shown in supplement Table S1.

#### Average time from the beginning of PC to patient death

Table 3 shows the average time from the beginning of PC to patient death in days. Results are similar to those of the utilization rates. Patients with ST received any form of PC earlier (*M* = 101, median = 50) than patients with HM (*M* = 91, median = 34). These differences were consistent across all types of care. PPC was initiated earliest in patients with ST (*M* = 11, median = 66) as well as in patients with HM (*M* = 110, median = 49).

**Table 3:** Average time from the beginning of PC to patient death (in days)

	Median (IQR)		*M* (SD)		Statistics Model 1		Statistics Model 2	
	ST	HM	ST	HM	Coefficient '*b*'	*p*	Coefficient '*b*'	*p*
Total palliative care	50 (15, 159)	34 (9, 138)	100.92 (111.21)	91.17 (113.28)	−9.7	<0.001	−3.2	0.064
Primary palliative care (PPC)	66 (20, 200)	49 (14, 200)	116.16 (116.29)	110.28 (120.62)	−5.9	0.007	1.9	0.4
Specialized palliative care (SPC)	27 (9, 74)	15 (6, 49)	59.60 (79.90)	48.09 (76.94)	−12	<0.001	−7.8	<0.001
Specialized palliative home care (SPHC)	29 (9, 77)	16 (6, 53)	61.48 (80.83)	49.93 (79.15)	−12	<0.001	−9.8	<0.001
Specialized palliative inpatient care (SPIC)	24 (8, 65)	15 (5, 47)	52.93 (71.71)	46.36 (72.51)	−6.6	<0.001	−1.8	0.4
Inpatient hospice care	15 (6, 35)	11 (5, 26)	32.46 (51.77)	27.82 (52.20)	−4.6	0.092	−8.7	0.002

PC types are not distinct, which means that a person might have utilized multiple PC types during his/her last year of life. *IQR* interquartile range. Coefficient '*b*' is the non-standardized coefficient of the regression. The coefficient indicates the difference between the two groups, ST and HM. In this context, it represents the estimate of how many days the ST and HM groups differ. *OR* odds ratio. Model 1 is a simple logistic regression. Model 2 includes age, sex, CCI, nursing care dependency, county rurality, year of death as covariates. Supplement Table S2 provides statistics of the covariates for Model 2

As shown in Table 3, all group differences between patients with ST and HM were significant in Model 1. When adjusting for covariates (Model 2) significant group differences were observed in all types of PC except for PPC. Further statistical details are presented in supplement Table S2.

### Analysis 2: Association of PC on the quality of end-of-life care in patients with ST and HM

Figure 1B shows the flowchart for analysis 2. As their utilization of PC started less than 30 days before death, 41,980 patients with ST (38% of those receiving PC) and 2321 patients with HM (47% of those receiving PC) were excluded. Thus, 153,008 individuals were included in Analysis 2.

The key characteristics of these patients are presented in Table 4. Patients with ST not receiving PC were older than those who received PC (for at least 30 days). In contrast, patients with HM who did not receive PC were younger than patients who received PC (for at least 30 days). In both patient groups, the proportion of women in the condition without PC was lower than in the condition with PC, with the highest proportion of women (52%) in patients with HM receiving PC. The CCI increased from no PC to PC in ST patients but remained relatively stable in HM patients.

**Table 4:** Sample characteristics analysis 2

Characteristic		ST		HM	
		noPC *N* = 87,540	PC *N* = 67,602	noPC *N* = 7,266	PC *N* = 2,612
Age (years)	Mean (SD)	79.55 (10.89)	74.48 (12.36)	77.06 (12.03)	78.94 (10.85)
Female	*n* (%)	36,460 (41.65%)	32,690 (48.36%)	3335 (45.91%)	1365 (52.27%)
Charlson Comorbidity Index (CCI)	Mean (SD)	8.35 (3.83)	10.36 (3.44)	6.55 (2.86)	6.49 (2.74)
Nursing care dependency at the time of death	*n* (%)	55,076 (62.92%)	59,912 (88.63%)	4167 (57.34%)	2274 (87.07%)
County rurality	Mean (SD)	0.18 (0.26)	0.20 (0.26)	0.19 (0.26)	0.19 (0.26)

**Fig. 2:**
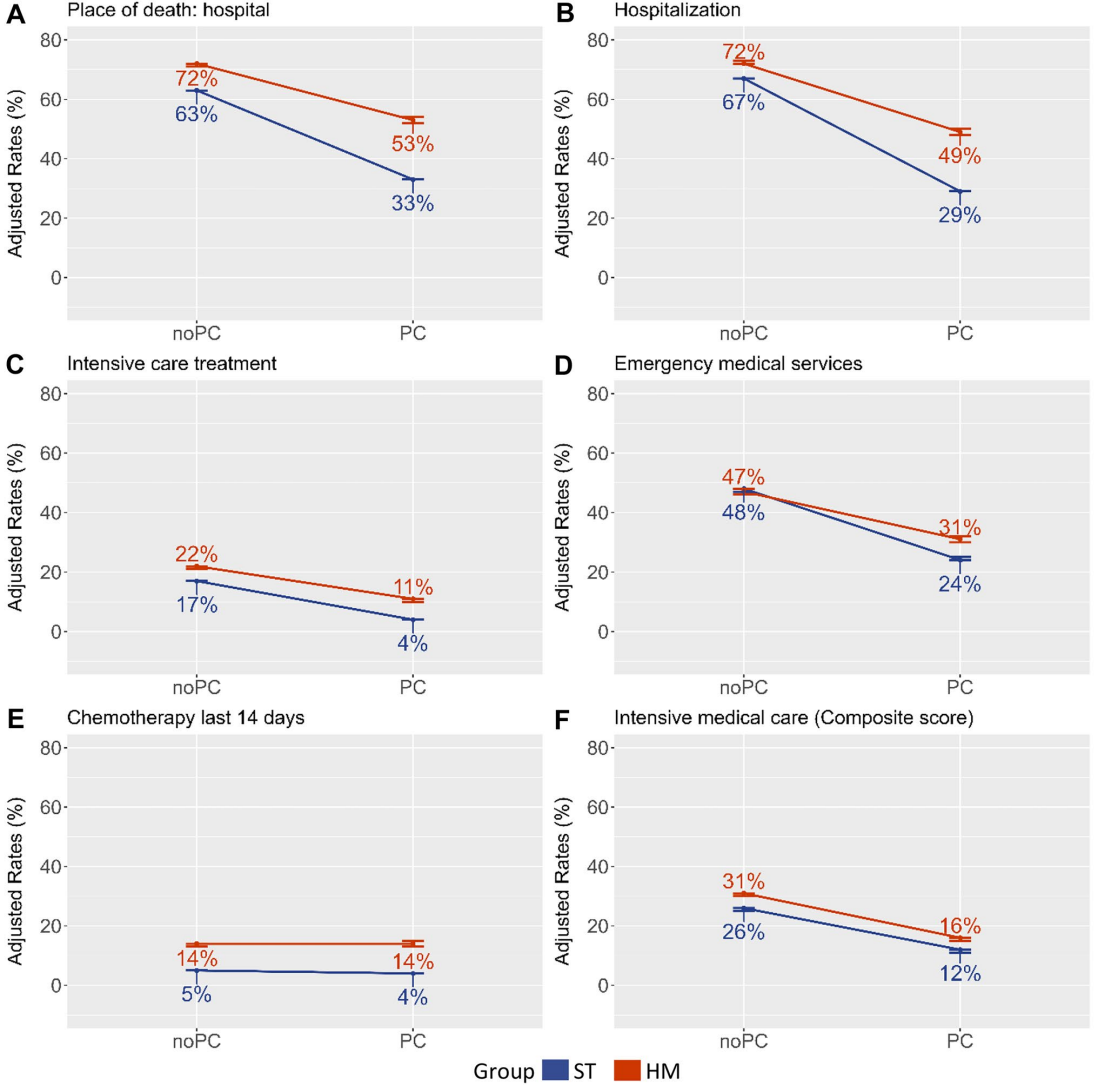
End-of-life quality indicators related to PC use and HM and ST patient groups

Figure 2A–F summarizes the results of the six indicators of the quality of end-of-life care. For all but one indicator (emergency medical services, *p* = 0.3), adjusted rates were on average higher for HM than for ST indicating worse quality of end-of-life care (all other *p* < 0.001). On average, patients who received PC were less likely to die in the hospital, to be hospitalized, to receive intensive care, to use emergency services, to receive chemotherapy in the last 14 days, and to receive Intensive medical care than those without PC (all *p* < 0.001). When we focused on whether the effect of PC differed between ST and HM, we found that patients with ST benefited more from PC than patients with HM on the following indicators. Significant interactions were found for Place of death hospital (*p* < 0.001), Hospitalization (*p* < 0.001), Intensive care treatment (*p* < 0.001), Emergency medical services (p < 0.001), and Chemotherapy in the last 14 days (*p* < 0.045). For the Intensive medical care indicator, the interaction term was not significant (*p* = 0.2), indicating that the effect of PC did not differ between ST and HM patients.

Detailed statistical results for all indicators can be found in supplement Table S3.

## Discussion

The presented analyses aimed to compare the utilization of different forms of PC, the time of the beginning of PC before death for different forms of PC, and the impact of PC on the quality of end-of-life care between patients with ST and patients with HM. Our main findings are: (1) Patients with HM had lower utilization rates and shorter time from PC beginning before death for all types of PC compared to patients with ST. (2) Patients with HM (after adjustment for covariates) had higher rates (i.e. worse outcomes) for almost all end-of-life quality indicators. (3) PC integration led to significant reductions in all six end-of-life quality indicators in both ST and HM patients. And (4) for almost all quality indicators, we found that ST patients benefited more from PC than HM patients.

The differences in utilization rates of PC, SPC and the beginning of PC between ST and HM patients are consistent with the patterns seen in other studies (Elliott et al. 2021; Howell et al. 2010b; Hui et al. 2014).

Our results differ from the findings of previous research. For instance, one study from the USA reported in 2014 higher rates of SPC at a tertiary cancer center (ST 47%, HM 33%) (Hui et al. 2014). In contrast, a US population-based study from 2001 to 2015 showed lower utilization in older blood cancer patients (ranging from 0.4 to 13.3%) (Rao et al. 2020). A 2011 systematic review by Howell et al. showed disparities across countries for patients with all cancers compared with those with HM (e.g., Hong Kong: 67 vs. 13%, USA: 59 vs. 21%, UK: 28 vs. 12%), with an overall OR of 0.46, suggesting a greater difference.

Looking at German PC utilization rates for all deceased patients from 2016 to 2019, an increase from 33.8 to 36.2% was observed, which were all lower than the rates for HM patients in our study (Ditscheid et al. 2023). This indicates that the integration of PC is progressing in Germany. At the same time, it can be seen that HM patients receive slightly higher rates of PC over the years compared to all other deceased patients, although differences between the ST and HM populations remain.

Few studies have thoroughly examined the time from begin of PC to patient death. A US study reported considerable variation in median time at a comprehensive cancer center, with 2 months for ST patients and 0.6 months for HM patients (Cheng et al. 2005). Another US study found a median time of 1.7 months for ST patients and 0.4 months for HM patients (Hui et al. 2014). Our data showed that in specialized PC, the median time is 27 days for ST and 15 days for HM patients. It should be emphasized that after adjustment there are no further significant differences in total PC, PPC, and SPIC. This highlights the importance of considering covariates when comparing cohorts and figures.

Reasons for differences in PC rates and timeframes in HM and ST patients between and even within countries are multifold. They may partly lie in the different organization of PC and its implementation in health care for HM and ST patients. The range of SPC services across Europe is reflected in the trend analysis by Arias-Casais et al. (2020). This shows that although the provision of SPC services has increased across Europe over the last 14 years, there are strong sub-regional disparities, with some PC services, such as home care teams or hospital support teams, missing. There are differences in PC utilization not only between countries, but also within countries. A recent study from Italy also shows that even within Italy, there are strong regional differences in the use of PC, despite the fact that the National Health System is available throughout the national territory (Ostan et al. 2024). An analysis of regional variation in PC utilization in Germany showed that differences in PC utilization cannot fully be explained by regional differences in the distribution of underlying diseases but rather by different financial structures of PC supply (Ditscheid et al. 2023).

Other reasons for the differences in PC rates and time frames are that most of the listed studies either included a specific patient group or specific diagnoses (Beaussant et al. 2018; Rao et al. 2020; Salas et al. 2022) or had smaller sample sizes (Burstein et al. 2023; Cheng et al. 2005; Howell et al. 2015; Hui et al. 2014). Some of the data in these studies are also more than 10 years old (Cheng et al. 2005; Howell et al. 2010b; Hui et al. 2014).

Despite intra- and international differences in PC supply and utilization, it seems that our overriding finding that PC is delivered less often and with a lower reduction of burdening therapies at the EOL in HM than in ST patients is more or less valid in every health care system, independent of the level of PC integration. On the contrary, system dependency is a much more decisive topic when it comes to the implementation of appropriate measures to improve the situation for HM patients in the future.

This study highlights further significant differences between patients with HM and those with ST, particularly regarding the integration of inpatient hospice care. Significantly fewer HM patients used inpatient hospice care, and the duration of care was much shorter. This pattern has also been identified in previous studies (Howell et al. 2010b; LeBlanc et al. 2018). An important reason for these differences is the restrictive practice of blood transfusion, which is a problematic barrier to appropriate end-of-life care in HM (LeBlanc et al. 2018). Blood transfusions are often no longer performed in inpatient hospices, making it difficult to refer HM patients to inpatient hospice care. Hematologic oncologists recognize the value of hospice care for HM patients, but the dependency on blood transfusions affects the decision to refer to hospice care and the timing of that referral, as revealed in a survey by Odejide et al. (Odejide et al. 2017).

According to other studies (Egan et al. 2020a; Howell et al. 2010a; Hui et al. 2014; Verhoef et al. 2020), our study shows that patients with HM have higher rates of burdening treatments in the last month of life compared to patients with ST. In our study, the differences are mostly smaller, e.g. for intensive care (Hui et al.: 39 vs. 8%, Egan et al.: 39 vs. 30%) (Egan et al. 2020a; Hui et al. 2014). Regarding chemotherapy, our results show similar rates as Egan et al. (12 vs. 5%) and higher rates compared to Hui et al. (43 vs. 14%)(Egan et al. 2020a; Hui et al. 2014).

Our results showed that patients with ST benefited more from PC than patients with HM on the indicator emergency medical services. A comparison with the more common emergency department (ED) indicator is difficult, because there are no billing figures for ED visits in Germany (Greiner et al. 2020). The rate of intensive medical care was higher in HM patients compared to acute leukemia patients in a French study by Salas et al. (rate: 12%) (Salas et al. 2022). This may be due to the fact that our study includes all types of hematologic malignancies, including severe diseases that may require more invasive measures. In addition, the disease course is less predictable.

Often treatment of various HM is associated with complications, including cytopenias, sudden bleeding, thromboembolic events, and severe anemia, as well as infections (Burstein et al. 2023; Salas et al. 2022). These complications often require hospitalization for monitoring and management of therapy (Egan et al. 2020b). In addition, many patients with HM continue their therapies until the end of life, resulting in additional side effects and requiring hospitalization for monitoring and administration of therapy (Burstein et al. 2023; Dasch et al. 2017; Hui et al. 2014).

Our results show a greater benefit from PC for patients with ST compared to those with HM. Differences in the integration of PC into treatment practice reflect this discrepancy. In contrast to ST, where PC is often part of treatment guidelines, its integration into HM treatment is incomplete (Mo et al. 2020). Several barriers have been identified by various authors that hinder the incorporation of PC into the care of patients with HM, including disease-specific, cultural, and systemic factors (El-Jawahri et al. 2020; Howell et al. 2010b; Odejide et al. 2016; Robbins-Welty et al. 2023; Wedding 2021). The complex disease course of HM often involves intensive treatments with significant morbidity and mortality, such as high-dose chemotherapy or CAR-T cell therapy. Uncertainty about prognosis complicates the transition from curative to PC and affects communication between hematologists and patients (Wedding 2021). Cultural barriers, such as the misconception that PC is only associated with end-of-life care, and the concerns of both patients and healthcare professionals also have an effect. Systemic barriers, including PC teams' lack of knowledge about HM and hospice services' limitations in providing blood products, are important factors. Zimmermann (2016) recommends the integration of PC for HM patients into routine care. In addition to the core elements of PC, such as symptom control, pain management, and psychological and spiritual care, other elements that are important for people with HM should be integrated. These include the possibility of blood transfusions for symptom control even in hospice, continuity of care by hematology teams, consideration of the unpredictability of the disease course, and precise definition of treatment guidelines (Wedding 2021; Zimmermann 2016). This requires disease-specific integration of PC, as each disease group has different needs, treatment paradigms, and outcomes (Robbins-Welty et al. 2023).

Considering the variable disease-specific trajectory of HM, routine implementation of PC screening may facilitate the identification of patients in need of PC. In addition to prognosis, screening for perceived patient distress (physical, psychological, social, and spiritual) in the form of patient-reported outcomes is particularly important (Bandieri et al. 2013; Gerlach 2020; Ramsenthaler et al. 2019).

There are various ideas for integrating PC into the treatment of HM, including trigger-based models, such as using events like an emergency department visit as a trigger for initiating PC (Verhoef et al. 2020), or early integration at the time of diagnosis, e.g. in patients with acute myeloid leukemia (El-Jawahri et al. 2021). There are approaches to integrate PC into the routine care of patients with HM, such as integration into a multiple myeloma PC clinic (Porta-Sales et al. 2017) or as a consultation unit in a bone marrow transplant unit (Selvaggi et al. 2014). The literature contains numerous calls to develop and to test effective models for integrating PC (Bandieri et al. 2013). Unfortunately, there are few studies on such models and a lack of analysis in real clinical practice. Furthermore, there is no consensus on the optimal approach for a given context (Tanzi et al. 2020), as this is also highly dependent on health care systems, availability of resources, and education and training in PC (Shaulov et al. 2022).

This study offers a comprehensive examination of PC utilization in Germany for HM compared to ST, benefiting from a robust sample size. It addresses gaps in prior research by differentiating various PC services and exploring the impact of PC on end-of-life care. However, several considerations limit the study. The outcome indicators used were developed and validated based on healthcare resource utilization in patients with ST. There is a lack of well-researched indicators specific to patients with HM (Beaussant et al. 2018; Earle et al. 2005; Earp et al. 2021). The constant changes and improvements in cancer treatment highlight the limitations of focusing on chemotherapy as an aggressive end-of-life treatment. It is, therefore, necessary that future studies take into account the constantly changing treatment environment and include targeted therapies, immune checkpoint inhibitors and other options as outcomes. Not all hospital care and treatment in the last days of life is inappropriate. HM patients, such as those with acute leukemia, often undergo intensive treatment with the primary goal of achieving a curative outcome. It is, therefore, important to be cautious when interpreting data on overtreatment at the end of life (Dasch et al. 2017). The study is based on data from a single health insurance provider, which limits generalizability to the entire German statutory health insurance population, despite standardization for age and sex. The heterogeneous group of patients with HM was combined into a single category. Differential analyses for each HM are still needed to determine the appropriate time point for PC. In addition, it is not possible to determine whether patients died of cancer or another disease.

## Conclusion

The results provide compelling evidence that hematologists should proactively and consistently provide PC to their patients or integrate PC into their care. To improve PC for patients with HM, it is critical to develop and rigorously test new models of PC integration. This process should start with the evaluation of screening strategies for PC needs and culminate in the structural integration of PC across various HM disease groups. In addition, a deeper understanding of existing barriers is essential to drive needed changes in a quality of life oriented PC for patients with HM.

### Supplementary Information

Below is the link to the electronic supplementary material.

Supplementary file1 (DOCX 53 KB)

## Data Availability

Access to data is only provided through data use agreement and via protected gateway to the scientific data warehouse of the health insurance fund BARMER. Further information about the data and access regulations are available from the corresponding author on request.
