# Supplementary material for: Utilization and quality of palliative care in patients with hematological and solid cancers: a population-based study
Source: J Cancer Res Clin Oncol. 2024 Apr 12;150(4):191. doi: 10.1007/s00432-024-05721-6 (PMC11014814; doi:10.1007/s00432-024-05721-6)
Supplement: Supplementary file 1 — Supplementary file1 (DOCX 53 KB) [file 432_2024_5721_MOESM1_ESM.docx]

**Supplement**

**Titel:** Utilization and Quality of Palliative Care in Patients with Hematological and Solid Cancers: A Population-Based Study

Cordula Gebel, Bianka Ditscheid, Franziska Meissner, Ekaterina Slotina, Isabel Kruschel, Ursula Marschall, Ullrich Wedding, Antje Freytag

**Table of contents**

**Supplement Table S1:** Palliative care utilization rates in the last year of life - Statistics for table 2 model 2 with covariates

**Supplement Table S2:** Average time from the beginning of PC to patient death *(in days)* - Statistics for table 3 model 2 with covariates

**Supplement Table S3:** Multiple logistic regression for indicators of end-of-life quality in relation to PC group and covariates

**Supplement Table S1:** Palliative care utilization rates in the last year of life - Statistics for table 2 model 2 with covariates

|  | PC | | PPC | | SPC | | SPHC | | SPIC | | Hospice | |
| --- | --- | --- | --- | --- | --- | --- | --- | --- | --- | --- | --- | --- |
| Characteristic | **OR** | **p-value** | **OR** | **p-value** | **OR** | **p-value** | **OR** | **p-value** | **OR** | **p-value** | **OR** | **p-value** |
| Group: HM (vs. ST) | 0.78 | <0.001 | 0.77 | <0.001 | 0.73 | <0.001 | 0.64 | <0.001 | 0.88 | <0.001 | 0.55 | <0.001 |
| Age (years) | 1.34 | <0.001 | 1.17 | <0.001 | 1.37 | <0.001 | 1.19 | <0.001 | 1.38 | <0.001 | 1.62 | <0.001 |
| Female | 0.96 | <0.001 | 0.98 | <0.001 | 0.94 | <0.001 | 0.96 | <0.001 | 0.95 | <0.001 | 0.96 | <0.001 |
| CCI | 1.12 | <0.001 | 1.08 | <0.001 | 1.12 | <0.001 | 1.08 | <0.001 | 1.13 | <0.001 | 1.06 | <0.001 |
| Nursing care dependancy at the time of death | 4.80 | <0.001 | 3.82 | <0.001 | 4.65 | <0.001 | 10.1 | <0.001 | 1.64 | <0.001 | 25.6 | <0.001 |
| County urality | 1.05 | 0.007 | 1.21 | <0.001 | 0.82 | <0.001 | 0.84 | <0.001 | 0.85 | <0.001 | 0.48 | <0.001 |
| Year of death | 1.00 | 0.2 | 0.96 | <0.001 | 1.03 | <0.001 | 1.04 | <0.001 | 1.01 | 0.003 | 0.99 | 0.024 |

Note: PC=Total palliative Care PPC = Primary palliative care; SPC = Specialized palliative care; SPHC = Specialized palliative home care; SPIC = Specialized palliative inpatient care; CCI = Charlson Comorbidity Index (CCI); OR = Odds Ratio

**Supplement Table S2:** Average time from the beginning of PC to patient death *(in days)* - Statistics for table 3 model 2 with covariates

|  | PC | | PPC | | SPC | | SPHC | | SPIC | | Hospice | |
| --- | --- | --- | --- | --- | --- | --- | --- | --- | --- | --- | --- | --- |
| Characteristic | **Coef:B** | **p-value** | **Coef:B** | **p-value** | **Coef:B** | **p-value** | **Coef:B** | **p-value** | **Coef:B** | **p-value** | **Coef:B** | **p-value** |
| Group: HM (vs.ST) | -3.2 | 0.064 | 1.9 | 0.4 | -7.8 | <0.001 | -9.8 | <0.001 | -1.8 | 0.4 | -8.7 | 0.002 |
| Age (years) | 3.6 | <0.001 | 2.9 | <0.001 | 6.2 | <0.001 | 6.2 | <0.001 | 5.9 | <0.001 | 2.8 | 0.002 |
| Female | -0.64 | <0.001 | -0.70 | <0.001 | -0.69 | <0.001 | -0.49 | <0.001 | -0.63 | <0.001 | -0.12 | 0.003 |
| CCI | 1.0 | <0.001 | 1.4 | <0.001 | 0.18 | 0.040 | -0.02 | 0.9 | 0.22 | 0.050 | -1.0 | <0.001 |
| Nursing care dependancy at the time of death | 25 | <0.001 | -0.83 | 0.5 | 39 | <0.001 | 23 | <0.001 | 39 | <0.001 | 18 | <0.001 |
| County rurality | 7.4 | <0.001 | 5.2 | 0.001 | -1.1 | 0.3 | 0.16 | >0.9 | -1.7 | 0.2 | -3.4 | 0.043 |
| Year of death | -0.94 | <0.001 | -0.56 | 0.062 | 0.90 | <0.001 | 1.4 | <0.001 | -0.21 | 0.4 | 0.43 | 0.15 |

Note: PC=Total palliative Care PPC = Primary palliative care; SPC = Specialized palliative care; SPHC = Specialized palliative home care; SPIC = Specialized palliative inpatient care; CCI = Charlson Comorbidity Index (CCI); OR = Odds Ratio

**Supplement Table S3:** Multiple logistic regression for indicators of end-of-life quality in relation to PC group and covariates

|  | Place of death:  hospital | | Hospitalization | | | Intensive care  treatment | | | Emergency medical services | | | Chemotherapy last 14 days | | | Intensive medical care | |
| --- | --- | --- | --- | --- | --- | --- | --- | --- | --- | --- | --- | --- | --- | --- | --- | --- |
| Characteristic | **OR***^1^* | **p-value** | **OR***^1^* | **p-value** | **OR***^1^* | | **p-value** | **OR***^1^* | | **p-value** | **OR***^1^* | | **p-value** | **OR***^1^* | | **p-value** |
| PC (vs. noPC) | 0.25 | <0.001 | 0.19 | <0.001 | 0.19 | | <0.001 | 0.35 | | <0.001 | 0.87 | | <0.001 | 0.36 | | <0.001 |
| HM (vs. ST) | 1.55 | <0.001 | 1.29 | <0.001 | 1.37 | | <0.001 | 0.97 | | 0.3 | 3.44 | | <0.001 | 1.32 | | <0.001 |
| Interaction PC (vs. noPC) * HM (vs. ST) | 1.61 | <0.001 | 1.88 | <0.001 | 2.08 | | <0.001 | 1.41 | | <0.001 | 1.20 | | 0.045 | 1.08 | | 0.2 |
| Female | 0.94 | <0.001 | 0.86 | <0.001 | 0.86 | | <0.001 | 0.78 | | <0.001 | 1.03 | | 0.2 | 0.79 | | <0.001 |
| Age (years) | 0.98 | <0.001 | 1.00 | <0.001 | 0.98 | | <0.001 | 1.00 | | <0.001 | 0.94 | | <0.001 | 0.97 | | <0.001 |
| CCI | 1.11 | <0.001 | 1.10 | <0.001 | 1.04 | | <0.001 | 1.01 | | <0.001 | 1.11 | | <0.001 | 1.03 | | <0.001 |
| Nursing care dependancy at the time of death | 0.33 | <0.001 | 0.60 | <0.001 | 0.43 | | <0.001 | 0.90 | | <0.001 | 0.45 | | <0.001 | 0.52 | | <0.001 |
| County rurality | 0.86 | <0.001 | 1.09 | <0.001 | 0.80 | | <0.001 | 1.03 | | 0.2 | 0.93 | | 0.11 | 1.04 | | 0.2 |
| Year of death | 1.01 | 0.018 | 1.00 | 0.7 | 1.03 | | <0.001 | 1.04 | | <0.001 | 1.01 | | 0.2 | 1.02 | | <0.001 |

Note: CCI = Charlson Comorbidity Index; OR = Odds Ratio
